# Not Your Typical Adenocarcinoma: A Case of Mesonephric Adenocarcinoma of the Cervix With Fibroblast Growth Factor Receptor 2 (FGFR2) Mutation

**DOI:** 10.7759/cureus.25098

**Published:** 2022-05-18

**Authors:** Sindhu Devarashetty, Suma Sri Chennapragada, Richard Mansour

**Affiliations:** 1 Hematology and Oncology, Louisiana State University Health Sciences Center-Shreveport, Shreveport, USA; 2 Internal Medicine, Louisiana State University Health Sciences Center-Shreveport, Shreveport, USA

**Keywords:** rare cancers, lenvatinib, fgfr2, gynaecologic oncology, mullerian and mesonephric ductal anomalies, invasive cervical cancer

## Abstract

Cervical cancer is one of the leading causes of cancer mortality in women. However, there have been great advances in its prevention and treatment. Nevertheless, there are certain rare forms of this cancer that are under-recognized, underreported, and have a paucity of evidence in terms of treatment. Mesonephric adenocarcinoma (AC) is one such rare disease, with less than 50 cases reported in the literature so far. We report a case of mesonephric AC of the cervix in a 73-year-old female who presented with abnormal vaginal bleeding. Our case is unique in that the patient had recurrence with lung metastases as well as fibroblast growth factor receptor 2 (FGFR2) mutation on genetic sequencing. She responded well to platinum-based chemotherapy and is currently on maintenance therapy with lenvatinib and bevacizumab. We aim to bring this patient’s disease course and treatment options chosen to the attention of the medical community as this is only the second reported case of mesonephric AC with FGFR2 mutation, and probably the first one to be treated with tyrosine kinase inhibitors and immunotherapy with a favorable response.

## Introduction

Mesonephric adenocarcinoma (AC) is a rare (<1% prevalence) form of AC, and it is believed to originate from the embryonic Wolffian remnants of the mesonephric duct [1]. The mesonephric duct is an embryonal structure that connects the cloaca to the primitive kidney. In males, it develops into the epididymis, vas deferens, and seminal vesicle. In females, however, due to the lack of anti-Mullerian hormone secretion, it regresses. However, its remnants can persist in the form of paroophoron, epoophoron, and Gartner’s duct [2]. Epoophoron is found between the ovary and the fallopian tissue. Paraophoron is found in the mesosalpinx, and Gartner’s ducts are found in the cervix and vagina. As many as 22% of adult uterine cervices have these remnants in their lateral cervical walls. When these remnants become pathological, they can develop into Gartner’s duct cysts, mesonephric hyperplasia, mesonephric AC, paraovarian cyst, and female adnexal tumors. Mesonephric AC is one such presentation, which is often seen in the cervix and very rarely in the uterine corpus, ovary, and vagina.

In this report, we present one such case of cervical mesonephric AC. We also engage in a detailed discussion of the treatment course to provide deeper insights into this disease process.

## Case presentation

Our patient was a 73-year-old Caucasian female who presented with vaginal bleeding and was found to have a 1.5 x 1.2 x 1.5-cm cervical mass on a CT scan. She then underwent a radical hysterectomy, and the pathology showed stage IB mesonephric AC of the cervix. Immunohistochemical staining showed that the tumor cells were focally positive for synaptophysin, p16, p53, CD10, and carcinoembryonic antigen, but negative for estrogen receptor. The patient went into remission for three years after receiving concurrent radiation therapy and cisplatin chemotherapy. A CT chest performed after three years showed multiple metastatic pulmonary lesions proven to be AC by biopsy. She then underwent six cycles of carboplatin, paclitaxel, and bevacizumab. Serial X-rays showed a stable disease process. Paclitaxel was stopped after two years, and bevacizumab maintenance therapy was continued for 2.5 years. CT scans were repeated and showed the progression of lung lesions. CancerPlex®/next generation sequencing (NGS) showed fibroblast growth factor receptor 2 (FGFR2) p.S252W mutation and high microsatellite instability (MSI). The patient was then started on lenvatinib and Keytruda as the MSI was high [3]. The patient is currently tolerating the treatment well with stable disease and is asymptomatic of her lung lesions.

## Discussion

Cervical cancer and subtypes

Cervical cancer is the third most common cancer and the fourth most common cause of cancer-related deaths in women as per the World Health Organization (WHO) statistics. In 2020, 604,000 new cases of cervical cancer and 342,000 deaths were reported across the world [4]. As early as the 1980s, the role of human papillomaviruses (HPV) in the causation of cervical cancer started to become evident as samples of biopsy specimens showed the presence of HPV 16 and HPV 18 [5]. Pathologically, squamous cell carcinoma (SCC), AC, and adenosquamous carcinoma (ASC) are the three major subtypes of cervical cancer identified. Of note, 90% of cervical cancers are of the SCC subtype, and AC of the cervix accounts for the rest along with other rare subtypes like neuroendocrine carcinoma, ASC, and anaplastic carcinoma to name a few.

Adenocarcinoma

Risk factors for AC include multiple sexual partners, young age at first sexual intercourse, obesity, use of oral contraceptives, and hormone replacement therapy. Most cases of AC of the cervix are HPV-related with HPV 16, 18, and 45 subtypes being implicated. However, unlike SCCs, a significant number of ACs are non-HPV related as well (10-15%) [6]. As per the WHO classification of tumors of the female reproductive organs 5th edition published in 2020, ACs are classified under glandular tumors and include the following categories: AC in situ of the uterine cervix - HPV-associated; AC of the uterine cervix - HPV-associated; AC in situ of the uterine cervix - HPV-independent; AC of the uterine cervix - HPV-independent, gastric type; AC of the uterine cervix - HPV-independent, clear cell type; AC of the uterine cervix - HPV-independent, mesonephric type; other ACs of the uterine cervix [1].

Mesonephric Adenocarcinoma 

Our review of the literature revealed that the mean age at diagnosis of this rare subtype of AC is 52 years whereas our patient was diagnosed at 73 years. The most common clinical presentation of this condition is abnormal vaginal bleeding. Other presentations include a cervical mass or an incidental diagnosis. Causative genetic mutations include GATA3+, p16-, ER-, and KRAS mutations [1]. However, FGFR mutations are rare in this type of cancer, and our patient is only the second such case reported in the literature [7]. It can be detected based on abnormal cervical cytology during routine screening but such detection occurs less frequently compared to SCC of the cervix. Diagnosis is made through specimens from cervical biopsy, curettage, or hysteroscopy. Of note, 70% of the patients are diagnosed at stage IB, with a recurrence rate of 32%, mean survival of 50 months, and a mean recurrence interval of 24 months [8]. Treatment depends on the stage of the disease but is mainly conducted through surgical methods like hysterectomy with or without bilateral salpingo-oophorectomy/pelvic node dissection. The role of chemotherapy and radiation are still being explored, and cases till now have been treated based on principles similar to those employed in other forms of cervical AC.

Treatment options

As of now, less than 50 cases of this rare type of cancer have been reported in the literature. Most of them have been treated with surgery and adjuvant radiation if diagnosed in the early stages. An institutional case series of 12 cases conducted by Xie et al. reported that adjuvant chemotherapy with carboplatin/paclitaxel led to a median progression-free survival of 12 months in these patients [9]. The recurrent disease has been treated with options like gemcitabine, carboplatin, and paclitaxel. Historically, these cancers have been very aggressive wherein one-third of the International Federation of Gynecology and Obstetrics (FIGO) stage 1 patients have developed recurrence even after curative resection, and one-fifth of the patients with stage 1B disease had a fatal course between one and nine years after the diagnosis [10]. Most patients died within one year of recurrence despite undergoing therapy. The incidence of lung metastasis is rare with only two cases reported in the literature so far [11,12]. However, the overall prognosis remains less favorable when compared to other cervical ACs.

Treatment in the current case

Our patient developed lung metastases after 36 months of initial therapy and responded well to carboplatin, paclitaxel, and bevacizumab for 30 months before progressing again. She is now doing well on a tyrosine kinase inhibitor (lenvatinib) and immunotherapy (pembrolizumab). Hence, this case is unique in having one of the longest courses of treatment and follow-up with newer therapies in the history of this rare disease process. We believe that bringing this patient’s disease course, its challenges, and treatment response to light is of vital importance toward a better understanding of this rare cancer. Our patient to date has had a course of around 66 months with a favorable response to novel treatment methods after recurrence. Our case also highlights the importance of repeat genetic testing when there is a recurrence as there is always a possibility of discovering new genetic mutations that can be targeted with novel therapies.

## Conclusions

Although the origin and histopathology of mesonephric AC differ from those of other cervical ACs, it is important to keep in mind that the same treatment principles still apply due to the paucity of pertinent evidence in the literature. However, we hope to highlight through this case report that the solution to discovering newer treatment options lies in advanced genetic testing for any mutations for which targeted therapies are currently available. FGFR2 mutation, though rare, is one of the targetable mutations in this cancer and needs to be tested for whenever possible. Similar continued efforts to report and collect further data on this rare disease process can help in devising more evidence-based treatments in the future.
